# Cost-effectiveness analysis of chlorhexidine-alcohol versus povidone iodine-alcohol solution in the prevention of intravascular-catheter-related bloodstream infections in France

**DOI:** 10.1371/journal.pone.0197747

**Published:** 2018-05-25

**Authors:** Franck Maunoury, Christian Farinetto, Stéphane Ruckly, Jeremy Guenezan, Jean-Christophe Lucet, Alain Lepape, Julien Pascal, Bertrand Souweine, Olivier Mimoz, Jean-François Timsit

**Affiliations:** 1 Statesia, Le Mans, France; 2 ICURE Search, Outcomerea, Paris, France; 3 University Hospital of Poitiers, Emergency Department and Pre-Hospital Care, Poitiers, France; 4 Infection Control Unit, AP-HP, Bichat University Hospital, Paris, France; 5 Department of Anaesthesiology and Critical Care Medicine, University Hospital of Lyon-Sud, Lyon, France; 6 Adults Intensive Care Unit, Ongoing Monitoring Unit, Clermont Ferrand University Hospital, Clermont-Ferrand, France; 7 Intensive Care Unit, Clermont Ferrand University Hospital, Clermont-Ferrand, France; 8 Paris Diderot University - Bichat University hospital - Medical and Infectious Diseases Intensive care unit, Paris, France; Public Library of Science, UNITED KINGDOM

## Abstract

**Objective:**

To perform a cost-effectiveness analysis of skin antiseptic solutions (chlorhexidine-alcohol (CHG) versus povidone iodine-alcohol solution (PVI)) for the prevention of intravascular-catheter-related bloodstream infections (CRBSI) in intensive care unit (ICU) in France based on an open-label, multicentre, randomised, controlled trial (CLEAN).

**Design:**

A 100-day time semi-markovian model was performed to be fitted to longitudinal individual patient data from CLEAN database. This model includes eight health states and probabilistic sensitivity analyses on cost and effectiveness were performed. Costs of intensive care unit stay are based on a French multicentre study and the cost-effectiveness criterion is the cost per patient with catheter-related bloodstream infection avoided.

**Patients:**

2,349 patients (age≥18 years) were analyzed to compare the 1-time CHG group (CHG-T1, 588 patients), the 4-time CHG group (CHG-T4, 580 patients), the 1-time PVI group (PVI-T1, 587 patients), and the 4-time PVI group (PVI-T4, 594 patients).

**Intervention:**

2% chlorhexidine-70% isopropyl alcohol (chlorhexidine-alcohol) compared to 5% povidone iodine-69% ethanol (povidone iodine-alcohol).

**Results:**

The mean cost per alive, discharged or dead patient was of €23,798 (95% confidence interval: €20,584; €34,331), €21,822 (€18,635; €29,701), €24,874 (€21,011; €31,678), and €24,201 (€20,507; €29,136) for CHG-T1, CHG-T4, PVI-T1, and PVI-T4, respectively. The mean number of patients with CRBSI per 1000 patients was of 3.49 (0.42; 12.57), 6.82 (1.86; 17.38), 26.04 (14.64; 42.58), and 23.05 (12.32; 39.09) for CHG-T1, CHG-T4, PVI-T1, and PVI-T4, respectively. In comparison to the 1-time PVI solution, the 1-time CHG solution avoids 22.55 CRBSI /1,000 patients, and saves €1,076 per patient. This saving is not statistically significant at a 0.05 level because of the overlap of 95% confidence intervals for mean costs per patient in each group. Conversely, the difference in effectiveness between the CHG-T1 solution and the PVI-T1 solution is statistically significant.

**Conclusions:**

The CHG-T1 solution is more effective at the same cost than the PVI-T1 solution. CHG-T1, CHG-T4 and PVI-T4 solutions are statistically comparable for cost and effectiveness.

This study is based on the data from the RCT from 11 French intensive care units registered with www.clinicaltrials.gov (NCT01629550).

## Introduction

Intravascular-catheter-related infections (CRI) are frequent life-threatening events in health care, but incidence can be decreased by improvements in the quality of care. In Europe, the incidence of CRBSIs ranges from 1 to 3.1 per 1,000 patient-days [1] and rates below 2 per 1,000 catheter-days are difficult to achieve in intensive care units (ICUs) [2,3] and for long-term intravenous catheters [4]. Optimization of skin antisepsis is essential to prevent short-term catheter-related infections. Through the CLEAN study [5], an open-label, multicentre, randomized, controlled trial (CLEAN, ClinicalTrials.gov Identifier: NCT01629550), we know that chlorhexidine–alcohol (CHG) would be more effective than povidone iodine–alcohol (PVI) as a skin antiseptic to prevent intravascular-catheter-related infections. The connection between CRBSI and mortality is a subject of a well-known methodological debate, driven by a major paper written by Soufir and Timsit et al. in 1999 [6].

For minimizing CRBSIs, a bundle of care combining sterile barrier precautions for insertion, appropriate antiseptic solution for skin antisepsis and line access, and immediate removal of avoidable catheters is recognized [7,8].

When the CLEAN study was carried out, the two solutions, CHG and PVI, were the ones most used in this indication without knowing with certainty if one was superior to the other; and the benefit of a debridement was not known and was still recommended in some countries like France. Hence four solution groups were considered in the study: CHG T1, CHG T4, PVI T1, and PVI T4. The objective of the study is to perform a cost-Effectiveness analysis (CEA) of these skin antiseptic solutions in the prevention of intravascular-catheter-related bloodstream infections in France from modeling techniques based on CLEAN database. To support the choice of the best antiseptic solution strategy from an ICU perspective, a decision-analytic model was performed.

## Methods

### Study design

Statistical analyses of both simulated and observed data from CLEAN database were performed. These analyses were based on a decision analysis model, and the adopted modeling approach for cost-effectiveness analysis complied with the guidelines of French National Authority for Health (Haute Autorité de Santé–HAS) [9]. The 100-day ICU-time non-homogeneous semi-Markovian model was based on ICU settings in France, and observed data of the CLEAN study. Model and data analyses were performed using Rstudio software (version 0.99.903–2009–2016 Rstudio, Inc.).

### Data collection

Clinical data of this study were from the CLEAN database delivered by the University Hospital of Poitiers. Adult ICU Patients from 11 French intensive-care units in five university hospitals and one general hospital were recruited [5]. This study compared the impact of the skin antiseptic CHG solution and of skin antiseptic PVI solution, both preceded (4-time, T4) or not (one time, T1) by skin cleaning with antiseptic detergent, on the rate of catheter-related infections (CRI). In the one-time (T1) procedure, the antiseptic was applied for at least 30 seconds and the catheter was then inserted once the work area was dry. In the four-time (T4) procedure, the skin was scrubbed first with antiseptic detergent for at least 15 seconds, rinsed with sterile water, and dried with sterile gauze. Antiseptic was then applied and the catheter was inserted once the work area was dry. The main objective of the RCT was to determine if the use of the CHG solution decreased CRI rates. Physicians and nurses were not masked to group assignment but microbiologists and outcome assessors were. The primary outcome was the incidence of catheter-related infections with chlorhexidine–alcohol (CHG) versus povidone iodine–alcohol (PVI) in the intention-to treat population. CRBSI rates in each of the four solution groups were also studied.

Cost data of this study were from literature and expert opinions and updated in Euro2016. The detailed sources and calculations (inputs) for base case input parameters for the cost analysis are shown in the “Base case input parameters for the cost analysis” sub-section. Simulated data of this study were from the cost-effectiveness model defined above through the Study Design sub-section, and detailed methodological approach and sources are available at the “Modeling and Statistical Analysis” sub-section.

### Study population

During the CLEAN RCT, consecutive (in order of arrival) adults (age ≥18 years) admitted to intensive-care units and requiring at least one of central-venous, haemodialysis, or peripheral arterial catheters were enrolled.

The investigators obtained written informed consent before study inclusion from competent (mentally capable) patients and at competence recovery from incompetent patients, according to French law. The study was approved by the ethics committee of the Poitiers University Hospital, France, based on French guidelines for prevention of catheter-related infection.

The study population included 2,349 patients assigned to PVI-T1 group (n = 588), PVI-T4 group (n = 580), CHG-T1 group (n = 587), CHG-T4 group (n = 594) [5]. The population without catheter is of 51 patients; as a consequence, a total of 2,298 catheterized patients were studied. Among these patients, the CRBSI population was of 34 patients (1.5% of the catheterized patients) including 22 men (65%) and 12 women (35%); 24 (71%) of these patients with CRBSI had at least one chronic disease (35% of the 2,298 catheterized patients in study); 11 (32%) of them died during the study, 8 (24%) of whom died in an intensive care unit. The sample sizes are reported from the database of the published CLEAN RCT [5], where Mimoz et al. (Lancet 2016) explained how subjects were excluded.

Among patients with CRBSI, there were 15 patients (44.1%) treated with PVI-T1 solution, 13 patients (38.2%) with PVI-T4 solution, 2 patients (5.9%) with CHG-T1 solution and 4 patients (11.8%) with CHG-T4 solution.

Patient and catheter characteristics, with or without CRBSI, are detailed in S1–S3 Tables.

### Endpoints

The primary clinical outcome was the incidence of CRBSI in patients assigned to CHG versus PVI as the skin antiseptic. The final health outcome of the cost-effectiveness analysis was the number of patients with CRBSIs avoided per 1000 patients (considering as a rare event) and the cost-effectiveness criterion was the ICER (Incremental Cost-Effectiveness Ratio) defined as the cost per patient with CRBSI avoided resulting from CHG skin antiseptic use. Indeed, regarding a cost-effectiveness analysis, the patient as the main statistical unit is more suited than the CRBSI event itself. If the studied strategy is more effective and less costly than the comparator, we can say that the studied strategy “dominates” the comparator.

### Modeling and statistical analysis

Markov models simulate the health trajectory of patients among distinct health states over time [10–13]. The main assumption of state-transition Markov models is that the next health state depends only on the present state and not on the sequence of events that preceded it. For an expected goodness of fit to CLEAN data, a multi-state semi-markovian model in continuous time was performed. Within this model, transition probabilities between states are time dependant and well suited to individual patient data (IPD) from CLEAN database. This type of modeling is suited to the context of ICU settings where progression of the patient cannot be considered as a chronic condition. Eight health states were considered (Table 1) in our cost-effectiveness model, four combining either occurrence, or no occurrence, of CRBSI, and the need, or no need, of a new central line (CT); one for contact dermatitis (AE: adverse event); one for changing to a neutral dressing (semipermeable transparent dressing) in case of dermatitis (skin reaction), and two absorbing states (death and discharge from the ICU).

**Table 1:** Health states defined from the CLEAN randomized controlled trial [5].

Health States	Definition
1. NoAE/noCRBSI/noCTnew*	Insertion of a first catheter, no contact dermatitis and no diagnosed CRBSI
**2. NoAE/noCRBSI/CTnew**	No contact dermatitis, no diagnosed CRBSI and a new catheter inserted
3. NoAE/CRBSI/noCTnew*	CRBSI diagnosed without neither contact dermatitis nor the need for inserting a new catheter
**4. NoAE/CRBSI/CTnew**	CRBSI diagnosed without contact dermatitis but the need for inserting a new catheter
5. AE/noCRBSI/noCTnew*	No diagnosed CRBSI, and no need for new catheter inserted but occurrence of contact dermatitis
**6. Dressing G+S**	Change to a neutral dressing strategy (semipermeable transparent dressing), if contact dermatitis occured
**7. Discharge**	Patient leaves the ICU alive
**8. Death**	Patient dies during the ICU stay

AE: Adverse event (dermatitis); CRBSI: Cathter-related bloodstream infection; CT: Catheter; G+S: Semipermeable transparent dressing.

*NoCTnew has 3 distinct hits: 1. No existing CT; 2. Existing CT, no removal of CT; 3. Removal of existing CT but no new CT. The mean cost of Markov states E1, E3 and E5 was a weighted mean cost considering the distribution of these hits within the CLEAN database.

**Fig 1:**
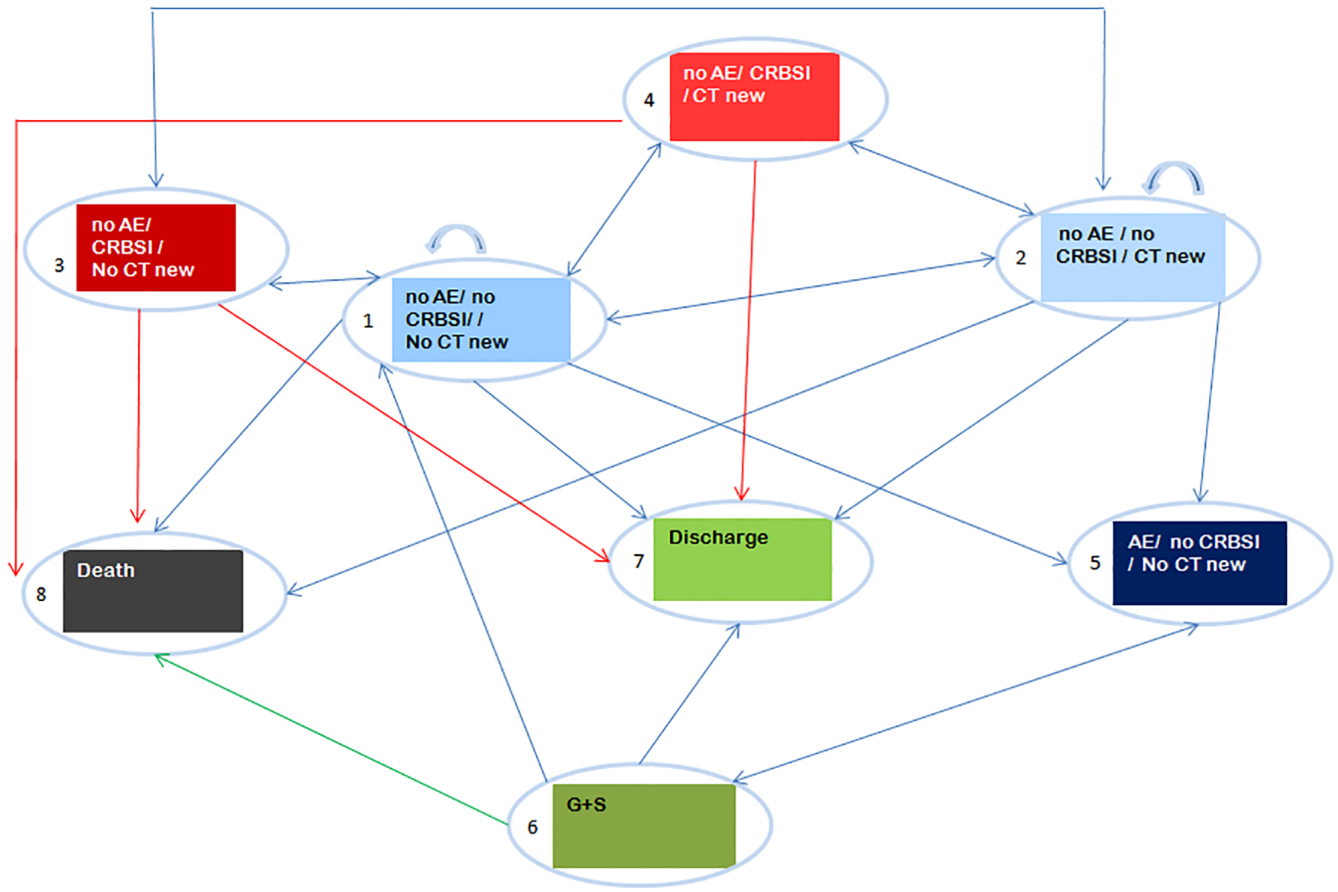
Observed model structure from CLEAN database (antiseptic skin CHG-solution T1/T4, antiseptic skin PVI-solution T1/T4)–Markov diagram. CHG: Chlorhexidine Alcohol, PVI: Povidone Alcohol, AE: Adverse event, CRBSI: Catheter-related bloodstream infection, CT: Catheter, G+S: Semipermeable transparent dressing.

The statistical unit of the study is the ICU patient within a time horizon of 100 days (including patients discharged alive from the ICU, alive but still in the ICU, or deceased during the ICU stay). Data was censored beyond 100 days because of the totality of CRBSI events were observed within this time period. The four estimated transition probability matrices were based on individual patient data from CLEAN database for each solution group (Supporting Information: S5 File). So, this simulation can be considered as a non-homogeneous Semi-Markov Chain (NH-SMC) simulation which takes into account time dependency of state transition, duration in each health state, and individual path of states through time. The possible transitions among health states are shown on the Markov diagram (Fig 1).

Instead of parametric Monte Carlo simulation, the *msm package* [14] allows to quantify uncertainty with non-parametric bootstrap methods for probabilistic sensitivity analysis and 95% confidence intervals (CI) calculations. To populate the model, data are specified as a series of observations, grouped by patient and sorted by increasing observational time from the patient entry in ICU. At minimum there should be a data frame with variables indicating:

- The time of the observation,
- The observed state of the process.

If the data do not also contain:

- The subject identification number (ID),

Then all the observations are assumed to be from the same subject. The subject ID does not need to be numeric, but data must be grouped by subject, and observations must be ordered by time within subjects.

**Fig 2:**
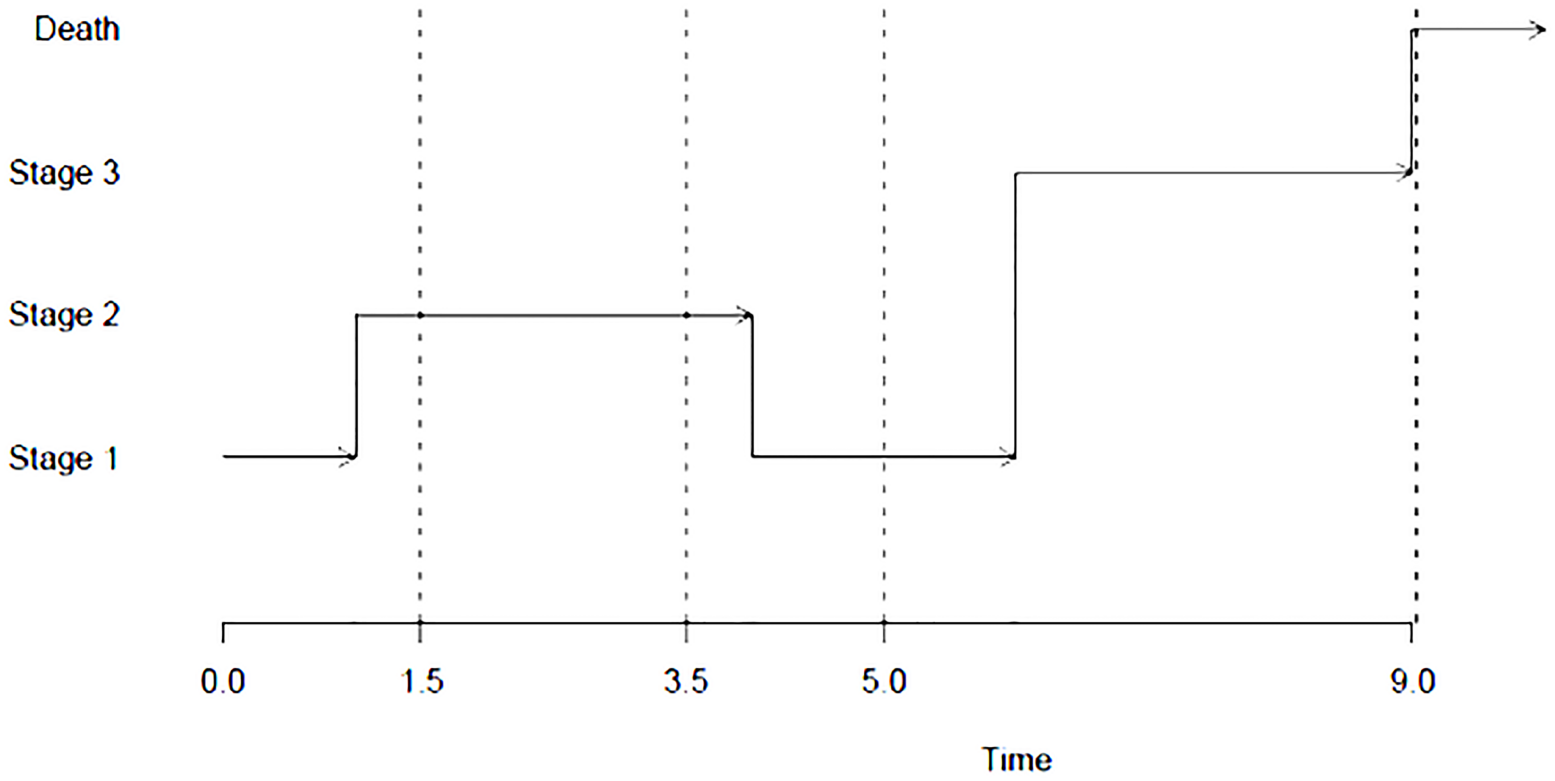
Evolution of a multi-state model. The process here is observed on four occasions (source: msm package from C. Jackson, 2016 [14]).

Fig 2 below shows how the non-homogeneous semi-Markov model we propose could work.

#### Main assumptions

1. The cost of a CRBSI event is independent from the outcome (survival or death or discharge): Statistical unit is the “global” patient;
2. Catheter colonization had no costs (after the diagnosis) or adverse outcomes (colonization has been initially excluded from the model because it’s not a “health-state”). The costs for diagnosis are negligible compared to costs related to additional ICU LOS due to CRBSI. The related costs to replace a catheter suspected to be colonized (and causing CRBSI) will be absorbed in the health-states CT new;
3. The estimated cost per ICU day at the University Hospital of Poitiers is estimated for each compared intervention;
4. If AE is dermatitis, the G+S (semipermeable transparent dressing) unit cost for CHG T1/T4 groups is identical to PVI T1/T4 groups;
5. Censored data for the "ICU Discharge" state, if the infection is observed two days after discharge from the ICU, we consider that this infection occurred the day of discharge from the ICU;
6. "G+S” State: Ability to integrate a potentially observable output "AE/no CRBSI/no CT New" (with probability for semipermeable transparent dressing). The considered AE is contact dermatitis.

#### Base case input parameters for the cost analysis

The base case analysis of the cost-effectiveness study has to be the most representative case of real life, taking into account current ICU settings in France, and according to the experts, literature and RCTs. For each patient group, the mean daily cost per ICU patient was partly based on the mean number of catheters and dressings per patient per day, which is shown in Table 2.

**Table 2:** Resources (catheters, dressings, antiseptic solutions) per patient—Statistical unit: The global patient with catheterization (alive, discharge or dead).

Statistics	CHG-T1	CHG-T4	PVl-T1 ti	PVl-T4
**Mean number of catheters/patient**	2,23	2,17	2,30	2,28
**Mean number of dressings/catheter**	3,85	3,90	3,97	4,06

The nursing time considered for 1 skin antiseptic application was included in the skin antiseptic solution unit cost.

The cost analysis was based on the following input parameters expressed in Euro 2016:

- Skin antiseptic solution costs per ICU patient per day (reported by University Hospital of Poitiers): CHG T1 solution, €3.45; CHG T4 solution, €4.85; PVI T1 solution, €1.98; PVI T4 solution, €3.53; semipermeable transparent dressing, €0.12 for each antiseptic solution.
- Cost of treating contact dermatitis per catheter: catheter removal, €29.11 [15]; four standard dressings, €27.68; catheter insertion, €116.90.
- Direct cost of treating CRBSI (mean/episode) [15]: €715.01.
- Cost per ICU patient per day [16]: €1,125.41.
- Additional ICU Length of stay (LOS) due to CRBSI: +11.77 days [5].
- Cost of added ICU LOS due to CRBSI: €13,246.05 (calculation).
- Cost per catheter change (assumption: 50% venous + 50% arterial) [15]: €117.02.
- Overall cost of one CRBSI (direct cost of treating one CRBSI + cost of additional ICU LOS due to CRBSI): €13,961.07 (calculation).

Direct costs for the treatment of CRBSIs were obtained from a micro-costing study [15]. ICU costs were based on an observational real life study [16] that assessed all resources consumed during a patient day in the ICU. This twenty-four hours multicentre prospective medico-economic study provides a complete overview and estimation of the average cost for medical and surgical ICUs in different hospital types in France: Hospitals (CH), University Hospitals (CHU) and Regional Hospitals (CHR). Twenty-two ICUs were selected randomly and all costs for 109 patients were estimated. For patients with CRBSI, an additional cost due to an extra ICU length of stay (LOS) was calculated.

#### Additional ICU length of stay due to CRBSI (input parameters)

We can estimate the additional ICU length of stay due to CRBSI based on individual patient data from CLEAN study through the following calculation: Mean ICU LOS in CRBSI patients–Mean ICU LOS in non-CRBSI patients. For a conservative scenario within this topic, as recommended by HAS [9] for the base case analysis, the potential comparator group was non-CRBSI patients in PVI T1. For this group, the mean difference in ICU LOS between CRBSI and non-CRBSI patients was of +11.77 days, that was very close to the published results of Schwebel et al. 2012 (11 days) [15].

#### Costs per patient per Markov state (input parameters)

The calculation of the cost for each Markov state (health state) per patient was done as follows, using the base case input parameters listed above:

- Skin antiseptic solution and dressing costs (including time needed per antiseptic solution, number of nurses involved, and materials used [15]) and cost per ICU day [16] were taken into account for health states 1–6;
- Cost of treating contact dermatitis [15]—(including catheter removal, four alternative dressings, and insertion of a new catheter) was taken into account only for health state 5;
- Cost of treatment of CRBSI [15] and additional ICU-LOS due to CRBSI [5] were taken into account for health states 3 and 4;
- Cost per catheter change (venous, arterial) [15] was taken into account for health states 2 and 4.

The estimated costs per Markov state per patient are shown in Table 3.

**Table 3:** Costs per patient per Markov state (input parameters).

Markov State	Costs for 1 patient CHG T1, Euro 2016	Costs for 1 patient CHG T4, Euro 2016	Costs for 1 patient PVI T1, Euro 2016	Costs for 1 patient PVI T4, Euro 2016
**1 NoAE/noCRBSI/noCTnew**	€1,188.09	€1,166.39	€1,143.48	€1,158.12
**2 NoAE/noCRBSI/CTnew**	€1,791.45	€1,772.48	€1,808.87	€1,827.83
**3 NoAE/CRBSI/noCTnew**	€15,149.15	€15,127.46	€15,104.54	€15,119.19
**4 NoAE/CRBSI/CTnew**	€15,752.52	€15,733.54	€15,769.94	€15,788.89
**5 AE/noCRBSI/noCTnew**	€1,575.76	€1,542.78	€1,543.31	€1,554.13
**6 Dressing G+S**	€1,155.65	€1,166.92	€1,144.02	€1,158.71
**7 Discharge**	-€	-€	-€	-€
**8 Death**	-€	-€	-€	-€

#### Final design of the cost-effectiveness model based on the CLEAN observed data (Supporting Information files: S1–S5 Files)

Results were dependent on the ICU time horizon and the duration of catheter exposure. In addition, the analysis of the CLEAN IPD showed that all CRBSI events occurred through a max time horizon of 60 days, so we decided to calculate the cost-effectiveness results based on these CLEAN IPD for a max 100-days time horizon in intensive care unit (ICU), taking into account all observed or potential CRBSI events and observed max duration of catheter exposure in each solution group. Accordingly, the NH-SMC model was performed on this basis, taking into account a 100-days time horizon in ICU (for base case analysis), and making assumption that all simulated patients were catheterized during this common time period for each of the four solution groups. Also, two simulations were performed: The first was a Non-Homogeneous Semi-Markov Chain simulation (NH-SMC) on “Global” patient sample. “Global” patient indicates a patient who could, during the ICU stay, be alive in the ICU, be alive and discharged of the ICU, or be died. The second was a NH-SMC simulation on “Alive” patient sample. “Alive” patient indicates a patient who could, during the ICU stay, be alive in the ICU, or be alive and discharged of the ICU. The patients who died were discarded of this simulation. The difference in cost-effectiveness result between these two simulations allows estimating the competitive impact of the mortality risk. As a consequence, cost-effectiveness results are shown in *Results* section for three distinct statistical analyses:

- The simulated “global” patient from the NH-SMC model based on CLEAN IPD,
- The simulated “alive” patient from the NH-SMC model based on CLEAN IPD,
- And the observed “global” patient from CLEAN IPD.

### Sensitivity analyses

The estimated transition probability matrix of the NH-SMC model was based on individual patient data from CLEAN study [5]. So, this simulation can be considered as a non-homogeneous Semi-Markov Chain Monte Carlo simulation which takes into account time dependency of state transition, duration in each state, and individual path of states through time. Instead of parametric Monte Carlo simulation, the *msm* package [14] allows to quantify uncertainty with non-parametric bootstrap methods. Then, bootstrap 95% confidence intervals were estimated for the transition probability matrix, the total length of stay in each state (estimate the expected total length of stay in each state for an individual in a given period of evolution of a multi-state model) [14], the expected number of visits in each state (estimate the expected number of visits in each state for an individual in a given period of evolution of a multi-state model) [14], and the mean cost per patient for a 100-days time horizon in ICU. The bootstrap method that has been adopted was that of the BCa algorithm which calculates confidence intervals using the Efron’s nonparametric bias- Corrected and accelerated (BCa) bootstrap method.

## Results

### The simulated “global” patient from the NH-SMC model based on CLEAN IPD

The expected total length of stay in each absorbing state for an individual between times 0 and 100 days was of 65.2 days (95%CI: [61.5; 68.5]) for state 7 (discharge) and 20.3 days (95%CI: [17.5; 23.7]) for state 8 (death) for ICU patients in CHG-T1 group. For the other compared groups, these results were of 68.7 days (64.9; 71.8) for state 7 (discharge) and 18.5 days (15.8; 21.9) for state 8 (death) for ICU patients in CHG-T4 group; 66.9 days (63.2; 70.1) for state 7 (discharge) and 19.5 days (16.6; 22.9) for state 8 (death) for ICU patients in PVI-T1 group; 66.6 days (62.9; 69.8) for state 7 (discharge) and 19.2 days (16.4; 22.9) for state 8 (death) for ICU patients in PVI-T4 group. These results showed that there was no statistical difference at 0.05 levels between groups for patient life expectancy based on a 100-day time horizon in ICU (between 79.7 and 81.5 days).

The cost-effectiveness results of base-case analysis for the simulated global Patient (alive, discharged, or dead) for ICU Time horizon of 100 days are shown in Table 4.

**Table 4:** Non-homogeneous Semi-Markov Chain simulation from observed data (CLEAN)–simulated global patient—ICU-time horizon: 100 days.

Strategy	PVI T1 (reference strategy)	PVI T4	CHG T1	CHG T4
**Mean Cost per patient (95%CI)**	€24,874 (€21,011; €31,678)	€24,201 (€20,507; €29,136)	€23,798 (€20,584; €34,331)	€21,822 (€18,635; €29,701)
**Effectiveness: Mean number of patients with CRBSI per 1000 patients (95%CI)**	22.37 (11.93; 54.82)	22.91 (11.68; 57.20)	2.59 (0.36; 16.79)	4.86 (1.26; 22.36)
**Difference in Cost per patient**	-	€-673	€-1,076	€-3,052
**Difference in Effectiveness per 1000 patients**	-	+0,54	-19,78	-17,51
**ICER / Dominance**	-	€1,246 (less costly, less effective)	Dominate PVI T1 (in mean: Less costly, more effective)	Dominate PVI T1 (in mean: Less costly, more effective)

ICER: Incremental Cost-Effectiveness Ratio = Difference in Cost / Difference in Effectiveness.

For a 100-day time horizon in ICU, the mean cost per patient varied between €21,822 (€18,635; €29,701) for CHG-T4 group, and €24,874 (€21,011; €31,678) for PVI-T1 group. The other groups were included in these bounds. The 95% confidence intervals (95%CI) show that difference in cost and difference in effectiveness are not statistically significant at 0.05 level (confidence intervals between strategies do overlap). As a consequence, for a 100-days time horizon in ICU setting, the cost-effectiveness of the four antiseptic solutions is statistically comparable if the simulated “Global” patient is considered for a 100-day time horizon in ICU.

### The simulated “alive” patient from the NH-SMC model based on CLEAN IPD

The expected total length of stay in the absorbing state 7 (discharge) for an individual between times 0 and 100 days in ICU was of 87.9 days (95%CI: [86.5; 89.2]) for ICU alive patients in CHG-T1 group. These results were of 87.8 days (86.3; 89.0) for ICU alive patients in CHG-T4 group; 86.8 days (85.3; 88.2) for ICU alive patients in PVI-T1 group; 86.2 days (84.7; 87.7) for ICU alive patients in PVI-T4 group. These results showed that there was no statistical difference at 0.05 levels between groups for ICU length of stay (between 12.1 and 13.8 days).

The cost-effectiveness results of base-case analysis for the simulated alive Patient (alive, discharged) for ICU Time horizon of 100 days are shown in Table 5.

**Table 5:** Non-homogeneous Semi-Markov Chain simulation from observed data (CLEAN)–Simulated alive patient—ICU-time horizon: 100 days.

Strategy	PVI T1 (reference strategy)	PVI T4	CHG T1	CHG T4
**Mean Cost per patient (95%CI)**	€24,874 (€21,011; €31,678)	€23,656 (€19,897; €29,744)	€22,557 (€18,879; €35,316)	€22,248 (€18,540; €32,096)
**Effectiveness: Mean number of patients with CRBSI per 1000 patients (95%CI)**	20.97 (10.29; 60.72)	19.23 (8.36; 65.06)	3.43 (0.48; 22.88)	6.15 (1.65; 30.48)
**Difference in Cost per patient**	-	€-1,218	€-2,317	€-2,626
**Difference in Effectiveness per 1000 patients (n CRBSI)**	-	-1,74	-17,54	-14,82
**ICER / Dominance**	-	Dominate PVI T1 (in mean: Less costly, more effective)	Dominate PVI T1 (in mean: Less costly, more effective)	Dominate PVI T1 (in mean: Less costly, more effective)

ICER: Incremental Cost-Effectiveness Ratio = Difference in Cost / Difference in Effectiveness.

For a 100-day time horizon in ICU, the mean cost per alive patient varied between €22,248 (€18,540; €32,096) for CHG-T4 group, and €24,874 (€21,011; €31,678) for PVI-T1 group. The other groups were included in these bounds. The 95% confidence intervals (95%CI) show that difference in cost and difference in effectiveness are not statistically significant at 0.05 levels (confidence intervals between strategies do overlap). As a consequence, for a 100-days time horizon in ICU setting, the cost-effectiveness of the four antiseptic solutions is statistically comparable if the simulated “Alive” patient is considered for a 100-day time horizon in ICU. The mortality, as a competitive risk, was not impactful on simulated results between groups.

### The observed “global” patient (alive, discharge or dead) from CLEAN IPD

From observed CLEAN individual patient data, the number of observed “global” patients with CRBSI (not simulated) per 1000 catheterized “global” patients is shown in Supporting Information S4 File.

The CHG-T1 solution prevents more patients from contracting a CRBSI than the other groups, with 3.49 (95%CI: 0.42; 12.57) patients with CRBSI per 1000 catheterized patients, versus 6.82 (1.86; 17.38) patients, 26.04 (14.64; 42.58) patients, and 23.05 (12.32; 39.09) patients for CHG-T4, PVI-T1, and PVI-T4 groups, respectively. The CHG-T1 solution was significantly more efficacious to prevent CRBSIs when compared to the PVI-T1 solution without any additional cost for the ICU (€21,927 (95%CI: €14,963; €28,458) per patient within a 100-day time horizon in ICU, versus €23,795 (€16,457; €30,662)); these costs were based on the observed cohort distribution (R output: $prevalences$‘observed percentages’) through Markov states (health states) over time (between 0 and 100 days in ICU), multiplied by the cost per patient matrix regarding each health state (Table 6).

**Table 6:** Cost-effectiveness results from observed data (CLEAN database)–Observed global patient—ICU-Time Horizon: 100 days.

Strategy	PVI T1 (reference strategy)	PVI T4	CHG T1	CHG T4
**Mean Cost per patient (95%CI)**	€23,795 (€16,457; €30,662)	€22,533 (€15,782; €28,838)	€21,927 (€14,963; €28,458)	€20,612 (€13,773; €27,042)
**Effectiveness: Mean number of patients with CRBSI per 1000 catheterized patients (95%CI)**	26.04 (14.64; 42.58)	23.05 (12.32; 39.09)	3.49 (0.42; 12.57)	6.82 (1.86; 17.38)
**Difference in Cost per patient**	-	€-1,262	€-1,868	€-3,183
**Difference in Effectiveness per 1000 catheterized patients**	-	-2,99	-22,55	-19,22
**ICER / Dominance**	-	Dominate PVI T1 (in mean: Less costly, more effective)	Dominate PVI T1 (in mean: Less costly, more effective)	Dominate PVI T1 (in mean: Less costly, more effective)

ICER: Incremental Cost-Effectiveness Ratio = Difference in Cost / Difference in Effectiveness.

The cost-effectiveness results for the observed “global” patient (not simulated), for each of the four compared groups and for a 100-day time horizon in ICU are shown in Table 6.

The 95% confidence intervals (95%CI) show that difference in cost was not statistically significant at 0.05 level (confidence intervals between strategies do overlap). On the opposite, the 95% confidence intervals (95%CI) show that difference in effectiveness between CHG-T1 and PVI-T1 groups was statistically significant at 0.05 level (confidence intervals between strategies do not overlap). As a consequence, for a 100-days time horizon in ICU setting, the cost-effectiveness of these 2 intervention groups (antiseptic solutions) was statistically not comparable if the observed “Global” patient was considered.

### Sensitivity analysis

Non-parametric bootstrap 95% confidence intervals (95%CI) were estimated for the transition probability matrix, the total length of stay in each Markov state, the expected number of visits in each state, and the mean cost per patient for a 100-days time horizon in ICU. The bootstrap method that has been adopted was that of the BCa algorithm which calculates confidence intervals using the Efron’s nonparametric bias- Corrected and accelerated (BCa) bootstrap method. From the NH-SMC model, the 95%CI lower and upper bounds of the three cost-effectiveness analyses (statistical units: simulated “global” patient, simulated “alive” patient, observed “global” patient) were shown in Tables 4–6. The CHG-T1 solution statistically dominates the PVI-T1 solution for the observed “global” patient cost-effectiveness analysis because of the non-overlapping nature of 95%CI regarding the effectiveness criteria that is the number of patients with CRBSI, without additional cost per patient (95%CI do overlap). For the two other cost-effectiveness analyses cited above (simulated “global” patient, and simulated “alive” patient), the 95%CI do overlap for costs and effectiveness.

## Discussion

The non-homogeneous multi-state semi-markovian model (NH-SMC) in continuous time is a suitable mathematical tool to be fitted to longitudinal data based on individual patient data (IPD) available in CLEAN database (University Hospital of Poitiers) [5]. The literature in this field frequently offers examples based on static decision tree models, used for both cost-effectiveness or cost-benefit studies [15,17,18], except for the latest Maunoury et al. 2015 publication on this topic [19]. The feature of the current simulation relates to the fact that it is based on daily real-life raw data, and not on published mean values found in the literature. The time-dependence addressed here (i.e. evolution of the risk of developing a CRBSI with increased catheterization time) corroborates that the non-homogeneous simulation approach is suitable considering the nature of the available data and the ICU settings (especially for severe patients with high mortality rate).

This NH-SMC model has some limitations. First, it was built on a single clinical study because it was the only RCT available with this particular product. Second, the cost-effectiveness analysis was based on a scenario specific to French ICUs, where the CRBSI rates are rather low (below 2 per 1,000 catheter-days [20,21]), as it is confirmed in this study. As a consequence, the NH-SMC model cannot be directly transposed to other settings or other countries with different CRBSI baseline rates. This transposition would require local individual data on time-dependent probabilities of transition among health states on a daily basis. Further studies involving other countries are needed to generalize our results and therefore our findings do not necessarily predict similar cost effectiveness of CHG antiseptic solutions in other countries or in specific patients’ subgroups.

We can see that the NH-SMC simulation did not capture all observed CRBSI events in CLEAN database because of the very low number of these observed events and so, the model under-estimates the counting of CRBSI events in each group. It is the reason why the differences in effectiveness (number of patients with CRBSI per 1000 catheterized patients on a 100-days ICU time horizon) were not statistically significant at 0.05 level, unlike the statistically significance was recorded from observational data (without simulation) between CHG-T1 and PVI-T1 groups. The simulated results were much closed to observational results but the estimated number of patients with CRBSI was lower than for the real raw data from CLEAN database.

According to the medical experts of our working group, there is no evidence to suggest that the infectious risk between each of the three types of catheters is different. A patient who has a central venous catheter (CVC) has almost always an arterial catheter (AC) and vice versa. A patient who has a hemodialysis catheter (HC) often has the other two: therefore it sounds impossible to do a catheter analysis. Moreover, according to the practical experience acquired in the RCT [20] and in another recently published study [22], the femoral and internal jugular routes were associated with a similar rate of infection in ICU patients (both studies), while the subclavian route presented a lower risk (2012 study [20] only had the subclavian route). However, even for subclavian CVCs, the use of CHG reduced the risk of infection. The corresponding subgroup analyses were reported in the electronic supplement of Timsit et al. study [20] accessible at http://www.atsjournals.org/doi/suppl/10.1164/rccm.201206-1038OC by selecting “timsit_ods.pdf” and illustrated by hazard ratios per subgroup (Supporting Information: S6 File): Hazard ratios (HR) and 95% confidence intervals (95%CI) represent the effect for each subgroup. The p-value tests the heterogeneity of CHG-gel dressing effect between subgroups. A p-value >0.05 indicates the absence of significant differences of effects between subgroups.

To explain the analysis of the average difference in cost between CHG-T1 and CHG-T4 groups over a 100-ICU days per infected or uninfected patient, deceased or not, out of ICU or not (e.g. "global patient” suited to real life), the analysis of deaths shows that the observed difference in death rate at 100 days between CHG-T1 (29.06%) and CHG-T4 (25.56%) did not cause the higher cost for CHG-T1 patients compared to CHG-T4 patients because there were more patients dying in the CHG-T1 group for 100 patients (%); so the average higher cost over 100 days for the global CHG-T1 patient versus CHG-T4 was not explained by the death absorbing state (state 8 valued at zero cost): it was therefore necessary to look for the explanation on the second absorbing state of the cost-effectiveness model (discharge from ICU). This underlined analysis showed that the higher average cost of the CHG-T1 patients was therefore well explained by the difference in percentage of state 7 (absorbing “discharge” state): The observed percentages of discharge between the two groups at 100 days was 73.73% and 70.03% for CHG-T4 and CHG-T1, respectively. In addition, there were more state7 patients (CHG-T1 388 Vs. CHG-T4 421) than state8 patients (CHG-T1 161 Vs. CHG-T4 146) in all patients, this explained why the average cost for one global patient was higher in the CHG-T1 group, compared to the CHG-T4 group over a 100-day time horizon, due to the zero cost valuation for the state7 and state8 absorbent states. The mean percentage of discharged patients on 100 days was higher in the CHG-T4 group than in the CHG-T1 group, which explained the higher cost of the CHG-T1 group.

If we compare the 100-days in ICU with the 30-days results, we can see the impact of CHG solutions regarding the mean cost per ICU global patient. Indeed, the mean cost per ICU patient was of €20,772 (95%CI: €18,663; €24,727), €19,581 (95%CI: €17,298; €24,200), €21,875 (95%CI: €19,078; €26,448), and €21,246 (95%CI: €18,615; €24,676) for CHG-T1, CHG-T4, PVI-T1 and PVI-T4, respectively. As for the 100-days time horizon in ICU, there was no difference statistically significant at 0.05 level, because of the overlapping nature of the four 95% confidence intervals. The mean costs per ICU global patient were lower for the CHG groups over 30 days than for the CHG groups over 100 days, as for the PVI groups, which is an expected result. But, differences in costs for each group between 100-days and 30-days time horizon in ICU lead us to conclude that CHG-T1 solution could induce savings owing to the highest difference in cost between the two time horizons studied, €-3,026 for CHG-T1 group, €-2,999 for PVI-T1 group, €-2,955 for PVI-T4 group, and €-2,241 for CHG-T4 group. As discharge from ICU and death are competitive risks for CRBSI events, and these two absorbent states are valued to zero cost, we can expect that CHG-T1 group will allow less deaths and/or less discharges from ICU, which is the main limitation of our 2-absorbent states study.

This study also has the non-technical limitation of being sponsored by industry (the BD Company). However, an external research organization (Statesia) was hired to handle independently the development of the simulation model and the data analysis to remove any possible bias. Non-BD authors have worked on the preparation of the manuscript, with the final version being approved by all non-BD authors prior to submission.

## Conclusions

According to the sensitivity analysis (bootstrap 95% confidence intervals) which addresses the level of uncertainty of the mean results, and the main results highlighted in this study, the CHG-T1 solution passed the test for cost-effectiveness even in the conservative scenario of very low CRBSI incidence. The CHG-T1 solution is significantly more efficient to prevent CRBSIs when compared to the PVI-T1 antiseptic solution without any additional cost for the ICU. As a consequence, from a cost-effectiveness point of view, we could recommend the routine use of this antiseptic solution for patients in intensive care units.

## Supporting information

S1 Table Characteristics of patients with CRBSI for each group. CRBSI: Catheter-Related Bloodstream Infection; CHG: Chlorhexidine Alcohol; PVI: Povidone Alcohol; T1/T4: 1/4 Time; ICU: Intensive Care Unit.
(DOCX)

S2 Table Patients with CRBSI: Age, SAPS and SOFA scores, length of hospital stay, length of ICU stay. CRBSI: Catheter-related bloodstream infection; mad: Mean absolute difference; sd: Standard deviation; Se: Standard error; SAPS: Simplified Acute Physiology Score; SOFA: Sequential Organ Failure Assessment score; ICU: Intensive care unit.
(DOCX)

S3 Table Patients without CRBSI: Age, SAPS and SOFA scores, length of hospital stay, length of ICU stay. CRBSI: Catheter-related bloodstream infection; mad: Mean absolute difference; sd: Standard deviation; Se: Standard error; SAPS: Simplified Acute Physiology Score; SOFA: Sequential Organ Failure Assessment score; ICU: Intensive care unit.
(DOCX)

S1 File Patient characteristics. (DOCX)

S2 File Designing optimal cost-effectiveness model from observed CLEAN individual patient data. (DOCX)

S3 File Influence of duration of catheter exposure on CRBSI occurrence. (DOCX)

S4 File Influence of intervention group on the number of patients with CRBSI per 1000 catheterized-patients. (DOCX)

S5 File Cost-effectiveness base case analysis (ICU time horizon: 100 days–Global patient). (DOCX)

S6 File Hazard ratios (HR) and 95% confidence intervals (95%CI) for each subgroup. (DOCX)
