# Supplementary material for: Cost-effectiveness analysis of chlorhexidine-alcohol versus povidone iodine-alcohol solution in the prevention of intravascular-catheter-related bloodstream infections in France
Source: PLoS One. 2018 May 25;13(5):e0197747. doi: 10.1371/journal.pone.0197747 (PMC5969756; doi:10.1371/journal.pone.0197747)
Supplement: S1 Table — CRBSI: Catheter-Related Bloodstream Infection; CHG: Chlorhexidine Alcohol; PVI: Povidone Alcohol; T1/T4: 1/4 Time; ICU: Intensive Care Unit. (DOCX) [file pone.0197747.s001.docx]

**S1 Table. Characteristics of patients with CRBSI for each group**

|  | **CRBSI Population n=34 (100%)** | **CHG-T1 n=2 (5.9%)** | **CHG-T4 n=4 (11.8%)** | **PVI-T1 n=15 (44.1%)** | **PVI-T4 n=13 (38.2%)** |
| --- | --- | --- | --- | --- | --- |
| **Men** | 22 (65%) | 0 (0%) | 3 (75%) | 10 (67%) | 9 (69%) |
| **At least one chronic disease** | 24 (71%) | 1 (50%) | 3 (75%) | 11 (73%) | 9 (69%) |
| **Metastatic cancer** | 0 (0%) | 0 (0%) | 0 (0%) | 0 (0%) | 0 (0%) |
| **Insulin-dependent diabetes mellitus** | 4 (12%) | 1 (50%) | 0 (0%) | 1 (7%) | 2 (15%) |
| **Solid organ transplant** | 0 (0%) | 0 (0%) | 0 (0%) | 0 (0%) | 0 (0%) |
| **Malignant hemopathy** | 1 (3%) | 0 (0%) | 1 (25%) | 0 (0%) | 0 (0%) |
| **Immunosuppressive** | 1 (3%) | 0 (0%) | 0 (0%) | 0 (0%) | 1 (8%) |
| **Heart failure** | 4 (12%) | 1 (50%) | 0 (0%) | 2 (13%) | 1 (8%) |
| **Renal failure** | 0 (0%) | 0 (0%) | 0 (0%) | 0 (0%) | 0 (0%) |
| **Respiratory failure** | 2 (6%) | 0 (0%) | 0 (0%) | 2 (13%) | 0 (0%) |
| **Invasive ventilation** | 22 (65%) | 2 (100%) | 2 (50%) | 10 (67%) | 8 (62%) |
| **Death** | 11 (32%) | 1 (50%) | 0 (0%) | 5 (33%) | 5 (38%) |
| **ICU death** | 8 (24%) | 0 (0%) | 0 (0%) | 4 (27%) | 4 (31%) |

CRBSI: Catheter-Related Bloodstream Infection; CHG: Chlorhexidine Alcohol; PVI: Povidone Alcohol; T1/T4: 1/4 Time; ICU: Intensive Care Unit.
