# Supplementary material for: Cost-effectiveness analysis of chlorhexidine-alcohol versus povidone iodine-alcohol solution in the prevention of intravascular-catheter-related bloodstream infections in France
Source: PLoS One. 2018 May 25;13(5):e0197747. doi: 10.1371/journal.pone.0197747 (PMC5969756; doi:10.1371/journal.pone.0197747)
Supplement: S2 Table — CRBSI: Catheter-related bloodstream infection; mad: Mean absolute difference; sd: Standard deviation; Se: Standard error; SAPS: Simplified Acute Physiology Score; SOFA: Sequential Organ Failure Assessment score; ICU: Intensive care unit. (DOCX) [file pone.0197747.s002.docx]

S2 Table. Patients with CRBSI: Age, SAPS and SOFA scores, length of hospital stay, length of ICU stay

| **Group: Patients with CRBSI** | | | | | | | | | |
| --- | --- | --- | --- | --- | --- | --- | --- | --- | --- |
| **Variables** | **n** | **mean** | **sd** | **median** | **mad** | **min** | **max** | **range** | **Se** |
| **Age** | 34 | 58.09 | 16.62 | 61.5 | 19.27 | 20 | 84 | 64 | 2.85 |
| **SAPS Score at baseline** | 34 | 46.76 | 19.58 | 46.5 | 20.02 | 17 | 89 | 72 | 3.36 |
| **SOFA Score at baseline** | 34 | 9.18 | 3.63 | 9.0 | 4.45 | 3 | 15 | 12 | 0.62 |
| **Length of ICU stay (days)** | 34 | 45.41 | 41.31 | 32.5 | 20.76 | 9 | 190 | 181 | 7.09 |
| **Length of hospital stay (days)** | 34 | 73.21 | 58.15 | 54.0 | 41.51 | 17 | 265 | 248 | 9.97 |

CRBSI: Catheter-related bloodstream infection; mad: Mean absolute difference; sd: Standard deviation; Se: Standard error; SAPS: Simplified Acute Physiology Score; SOFA: Sequential Organ Failure Assessment score; ICU: Intensive care unit.
