# Supplementary material for: Cost-effectiveness analysis of chlorhexidine-alcohol versus povidone iodine-alcohol solution in the prevention of intravascular-catheter-related bloodstream infections in France
Source: PLoS One. 2018 May 25;13(5):e0197747. doi: 10.1371/journal.pone.0197747 (PMC5969756; doi:10.1371/journal.pone.0197747)
Supplement: S3 Table — CRBSI: Catheter-related bloodstream infection; mad: Mean absolute difference; sd: Standard deviation; Se: Standard error; SAPS: Simplified Acute Physiology Score; SOFA: Sequential Organ Failure Assessment score; ICU: Intensive care unit. (DOCX) [file pone.0197747.s003.docx]

**S3 Table. Patients without CRBSI: Age, SAPS and SOFA scores, length of hospital stay, length of ICU stay**

| **Group: Patients without CRBSI** | | | | | | | | | |
| --- | --- | --- | --- | --- | --- | --- | --- | --- | --- |
| **Variables** | **n** | **mean** | **sd** | **median** | **mad** | **min** | **max** | **range** | **Se** |
| **Age** | 2264 | 62.14 | 15.45 | 64.0 | 14.83 | 18 | 93 | 75 | 0.32 |
| **SAPS Score at baseline** | 2264 | 52.50 | 20.14 | 51.0 | 20.76 | 0 | 125 | 125 | 0.42 |
| **SOFA Score at baseline** | 2264 | 8.86 | 3.70 | 9.0 | 4.45 | 0 | 20 | 20 | 0.08 |
| **Length of ICU stay (days)** | 2264 | 14.35 | 20.28 | 8.0 | 7.41 | 1 | 415 | 414 | 0.43 |
| **Length of hospital stay (days)** | 2264 | 33.24 | 37.67 | 22.0 | 20.76 | 1 | 429 | 428 | 0.79 |

CRBSI: Catheter-related bloodstream infection; mad: Mean absolute difference; sd: Standard deviation; Se: Standard error; SAPS: Simplified Acute Physiology Score; SOFA: Sequential Organ Failure Assessment score; ICU: Intensive care unit.
