# Supplementary material for: Cost-effectiveness analysis of chlorhexidine-alcohol versus povidone iodine-alcohol solution in the prevention of intravascular-catheter-related bloodstream infections in France
Source: PLoS One. 2018 May 25;13(5):e0197747. doi: 10.1371/journal.pone.0197747 (PMC5969756; doi:10.1371/journal.pone.0197747)
Supplement: S1 File — (DOCX) [file pone.0197747.s004.docx]

**S1 File: CRBSI and non-CRBSI Patient characteristics**

We have detailed statistical characteristics of the quantitative variables: age, SAPS score, SOFA score for the population with CRBSI, and the subgroups assigned to the four alcoholic solutions.

According to S2 Table, for the population with CRBSI, the median age was of 61.5 years, SAPS and SOFA median scores at baseline were of 46.5 and 9.0, respectively. These scores should be linked to the observed death rate of 32%.

The median length of stay (LOS) in ICU and hospital were 32.5 and 54.0 days, respectively. The maximum LOS in ICU and hospital were of 190 and 265 days, respectively.

For comparison, the same statistics were calculated (S3 Table) for ICU patients without CRBSI. The median LOS in ICU and hospital were lower than for patients with CRBSI: 8 and 22 days, respectively. However, the maximum length of ICU and hospital stays was higher: 415 and 429 days, respectively.
