# Supplementary material for: Cost-effectiveness analysis of chlorhexidine-alcohol versus povidone iodine-alcohol solution in the prevention of intravascular-catheter-related bloodstream infections in France
Source: PLoS One. 2018 May 25;13(5):e0197747. doi: 10.1371/journal.pone.0197747 (PMC5969756; doi:10.1371/journal.pone.0197747)
Supplement: S2 File — (DOCX) [file pone.0197747.s005.docx]

**S2 File: Designing optimal cost-effectiveness model from observed CLEAN individual patient data**

**Influence of skin antiseptic solution on CRBSI prevention**

Thanks to the randomization from the CLEAN RCT [5], patients studied in the 4 solution groups (CHG-T1, CHG-T4, PVI-T1, PVI-T4) are recognized as comparable.

Table 1: Overall Population - Age, severity scores and lengths of stay (ICU and overall hospitalization)

| n=2,349 | | | | | | | |
| --- | --- | --- | --- | --- | --- | --- | --- |
| Variable | n | Mean | Sd | Median | Min | Max | Range |
| Age (years) | 2349 | 62.03 | 15.49 | 64 | 18 | 93 | 75 |
| SAPS score at baseline | 2349 | 52.29 | 20.21 | 51 | 0 | 125 | 125 |
| SOFA score at baseline | 2349 | 8.81 | 3.73 | 9 | 0 | 20 | 20 |
| ICU Length of stay (days) | 2349 | 14.71 | 20.94 | 8 | 1 | 415 | 414 |
| Hospitalization Length of stay (days) | 2349 | 33.57 | 38.02 | 22 | 1 | 429 | 428 |

Table 2: PVl-AC 1-time group - Age, severity scores and lengths of stay (ICU and overall hospitalization)

| n=588 | | | | | | | |
| --- | --- | --- | --- | --- | --- | --- | --- |
| Variable | n | mean | sd | median | min | max | range |
| Age (years) | 588 | 60.78 | 15.32 | 63 | 19 | 93 | 74 |
| SAPS score at baseline | 588 | 50.84 | 19.50 | 48 | 0 | 120 | 120 |
| SOFA score at baseline | 588 | 8.78 | 3.68 | 9 | 1 | 19 | 18 |
| ICU Length of stay (days) | 588 | 14.98 | 17.00 | 9 | 1 | 140 | 139 |
| Hospitalization Length of stay (days) | 588 | 33.35 | 34.77 | 22 | 2 | 230 | 228 |

Table 3: PVl-AC 4-time group - Age, severity scores and lengths of stay (ICU and overall hospitalization)

| n=580 | |  |  |  |  |  |  |
| --- | --- | --- | --- | --- | --- | --- | --- |
| Variable | n | mean | sd | median | min | max | range |
| Age (years) | 580 | 62.76 | 15.52 | 64 | 18 | 92 | 74 |
| SAPS score at baseline | 580 | 52.02 | 19.73 | 51 | 6 | 118 | 112 |
| SOFA score at baseline | 580 | 8.53 | 3.75 | 8 | 0 | 20 | 20 |
| ICU Length of stay (days) | 580 | 14.92 | 21.69 | 8 | 1 | 190 | 189 |
| Hospitalization Length of stay (days) | 580 | 32.58 | 35.64 | 21 | 1 | 265 | 264 |

Table 4: CHX-alc 1-time group - Age, severity scores and lengths of stay (ICU and overall hospitalization)

| Groupe : CHX-alc 1 time n=587 | |  |  |  |  |  |  |
| --- | --- | --- | --- | --- | --- | --- | --- |
| Variable | n | mean | sd | median | min | max | range |
| Age (years) | 587 | 62.49 | 14.99 | 64 | 18 | 90 | 72 |
| SAPS score at baseline | 587 | 53.03 | 20.88 | 51 | 10 | 125 | 115 |
| SOFA score at baseline | 587 | 8.98 | 3.79 | 9 | 0 | 20 | 20 |
| ICU Length of stay (days) | 587 | 15.11 | 25.38 | 8 | 1 | 415 | 414 |
| Hospitalization Length of stay (days) | 587 | 33.19 | 37.66 | 24 | 1 | 416 | 415 |

Table 5: CHX-alc 4-time group - Age, severity scores and lengths of stay (ICU and overall hospitalization)

| n=594 | |  |  |  |  |  |  |
| --- | --- | --- | --- | --- | --- | --- | --- |
| Variable | n | mean | sd | median | min | max | range |
| Age (years) | 594 | 62.11 | 16.08 | 64 | 18 | 88 | 70 |
| SAPS score at baseline | 594 | 53.24 | 20.65 | 52 | 6 | 109 | 103 |
| SOFA score at baseline | 594 | 8.94 | 3.68 | 9 | 1 | 19 | 18 |
| ICU Length of stay (days) | 594 | 13.86 | 18.81 | 8 | 1 | 282 | 281 |
| Hospitalization Length of stay (days) | 594 | 35.13 | 43.38 | 22 | 1 | 429 | 428 |

Indeed they were; the mean age of patients was of 62 years (standard deviation, sd: 15,5 years), the mean Simplified Acute Physiology Score (SAPS) to predict hospital mortality was of 52.3 (sd: 20.2) at baseline, the mean Sequential Organ Failure Assessment severity score (SOFA) predicting ICU mortality based on lab results and clinical data [17] was of 8.8 (sd: 3.7) at baseline, the mean ICU length of stay was of 14.7 days (sd: 20.9), and the mean hospital length of stay was of 33.6 days (sd: 38). Among these patients, 2 patients over 572 (0.35%), 4 patients over 586 (0.68%), 15 patients over 576 (2.60%), and 13 patients over 564 (2.30%) were diagnosed with CRBSI in CHG-T1 group, CHG-T4 group, PVI-T1 group, and PVI-T4 group, respectively. These CRBSI rates were statistically significant at 0.05 level (bilateral Fisher Exact p-value: 0.0011). Then, to estimate what solution groups were statistically different, we carried out a bilateral Fisher exact post hoc test with Bonferroni correction. The differences in rate between CHG-T1 group and PVI-T1/T4 groups were statistically significant, with p-values of 0.013 and 0.022, respectively.
