# Supplementary material for: Cost-effectiveness analysis of chlorhexidine-alcohol versus povidone iodine-alcohol solution in the prevention of intravascular-catheter-related bloodstream infections in France
Source: PLoS One. 2018 May 25;13(5):e0197747. doi: 10.1371/journal.pone.0197747 (PMC5969756; doi:10.1371/journal.pone.0197747)
Supplement: S3 File — (DOCX) [file pone.0197747.s006.docx]

**S3 File: Influence of duration of catheter exposure on CRBSI occurrence**

The results did consider the duration of catheter exposure in each group. The Kruskal-Wallis test was significant on this topic (p-value: 0); in order to identify which groups were responsible for null hypothesis (H0) rejection, a post hoc multiple comparison procedure (Dunn method) was used. A significant statistical difference at 0.05 level was observed between PVI-T1 and PVI-T4 groups (p-value: 0.0008) with a mean duration of catheter exposure of 36.8 and 115.3 days, respectively. For CHG-T1 and CHG-T4 groups, these durations were of 51.0 and 57.2 days, respectively. We decided to carry out a logistic regression of the probability of being in the CRBSI state as a function of the duration of catheter exposure.

R output:

glm(formula = CRBSI ~ durcat1 + groupe, family = "binomial",
    data = catheter)
Deviance Residuals:
    Min       1Q    Median  3Q      Max
-0.5732  -0.1334  -0.1148  -0.0620   3.5198

Coefficients:
                      Estimate Std. Error z value Pr(>|z|)
(Intercept)           **-7.09404**    0.7386   -9.604  **< 2e-16 *****durcat1                **0.06010**    0.01563   3.844 **0.000121 *****
groupeCHX-alc 4 temps  0.71064    0.86752    0.819 0.412695
groupePVl-AC 1 temps   2.00576    0.75451    2.658 **0.007852 ****
groupePVl-AC 4 temps   1.95563    0.75760    2.581 **0.009842 ****
---
Signif. codes:  0 ‘***’ 0.001 ‘**’ 0.01 ‘*’ 0.05 ‘.’ 0.1 ‘ ’ 1

(Dispersion parameter for binomial family taken to be 1)

    Null deviance: 419.28  on 5158  degrees of freedom
Residual deviance: 391.21  on 5154  degrees of freedom
AIC: 401.21

Number of Fisher Scoring iterations: 9

                               OR        2.5 %        97.5 %
**(Intercept)            0.000830034 0.0001326714   0.002787264
durcat               1.061941609 1.0275359076 1.093113469**groupeCHX-alc 4 temps 2.035283327 0.3960680083 14.713128119
**groupePVl-AC 1 temps  7.431768841 2.0879005968 47.264536162
groupePVl-AC 4 temps  7.068363752 1.9667557016 45.113275832**

Based on this logistic model, duration of catheter exposure (p-value: 0.0001; estimated odds ratios (OR): 1.06 (95% confidence interval (CI): [1.03; 1.09]) and solution group (p-value of 0.0078 for PVI-T1 group; OR: 7.43 (95%CI: [2.09; 47.26]), and p-value of 0.0098 for PVI-T4 group; OR: 7.07 (95%CI: [1.97; 45.11]) were significant variables to explain CRBSI occurrence. It can be concluded that the risk of contracting a CRBSI increases with the duration of catheter exposure and type of skin antiseptic solution.

As a consequence, this led to estimate the influence of skin antiseptic solution on incidence of CRBSI per 1000 catheter-days. Based on CLEAN data provided by the University Hospital of Poitiers, the mean number of CRBSI per 1000 catheter-days was of 0.18 (95%CI: [0.00; 0.65]), 0.38 (95%CI: [0.10; 0.95]), 1.33 (95%CI: [0.73; 2.12]), and 1.31 (95%CI: [0.74; 2.13]) for CHG-T1, CHG-T4, PVI-T1, and PVI-T4 groups, respectively. We have performed simulations of this statistic using non-parametric bootstrap from R software [14]. The bootstrap method that has been adopted is that of the BCa algorithm which calculates confidence intervals using the Efron's nonparametric bias- Corrected and accelerated (BCa) bootstrap method. We noted that the number of observed CRBSI per 1000 catheter-days was lower for the CHG-T1 and CHG-T4 alcohol solutions (0.185; 0.382) than for the PV1-T1 and PV1-T4 solutions (1.332; 1.306). The 95% confidence interval estimated by nonparametric bootstrap indicates that there was a statistically significant difference at a 0.05 level between CHG-T1 group and PVI-T1/4 groups (the upper limit of the 95% confidence interval in the CHG-T1 group is lower than the lower limit of the confidence intervals in the PVI-T1/4 groups (the confidence intervals do not overlap). On the contrary, there was no statistically significant difference between CHG-T1 and CHG-T4 groups, but CHG-T1 seems to further protect the patient from CRBSI (the lower bound of 95% confidence interval was equal to 0 for this group).

*In fine*, to propose an optimal type of model for cost-effectiveness analysis, we tested whether differences in proportions of deaths observed among CRBSI patients were statistically significant using a two-sided proportional test. For patients with CRBSI, since the number of cells was less than 5 in several cells, the usual test of Chi² could not be applied and we opted to use Fisher's exact test. For patients without CRBSI, the two-sided chi-square test was chosen because the cell counts were sufficiently large (> 5). For these two specific populations, the p-value being greater than 0.05 we concluded that differences in proportions of deaths among CRBSI (p-value: 0.5253) and non-CRBSI (p-value: 0.5779) patients in the four intervention groups were not statistically significant at the 0.05 level.
