# Supplementary material for: Cost-effectiveness analysis of chlorhexidine-alcohol versus povidone iodine-alcohol solution in the prevention of intravascular-catheter-related bloodstream infections in France
Source: PLoS One. 2018 May 25;13(5):e0197747. doi: 10.1371/journal.pone.0197747 (PMC5969756; doi:10.1371/journal.pone.0197747)
Supplement: S4 File — (DOCX) [file pone.0197747.s007.docx]

**S4 File: Influence of intervention group on the number of patients with CRBSI per 1000 catheterized-patients**

We used the Exact Binomial Test for 95% confidence interval calculations. This statistical test performs an exact test of a simple null hypothesis about the probability of success in a Bernoulli experiment. This guarantees that the confidence level is at least *conf.level*, but in general does not give the shortest-length confidence intervals. Consequently, this estimation is quite conservative and can show that the difference in proportion of number of patients with CRBSI per 1000 catheterized patients between groups is statistically significant (if 95%CI do not overlap). The number of patients with CRBSI per 1000 catheterized patients is shown in Table 7; we can see that the difference in proportion of number of patients with CRBSI per 1000 catheterized patients in CHG-T1 (mean: 3.49, 95%CI: [0.42; 12.57]) and PVI-T1 (mean: 26.04, 95%CI: [14.64; 42.58]) groups is statistically significant at 0.05 level (95%CI do not overlap).

Number of observed patients with CRBSI per 1000 catheterized patients (from CLEAN database): Statistical unit is the global patient with catheterization (alive, discharge or dead)

| **Group** | **Number of patients with CRBSI per n patient in each group** | **Number of patients with CRBSI per 1000 catheterized patients**  **Mean [95%CI]** |
| --- | --- | --- |
| **CHG-T1** | 2/572 (0.349%) | 3.49 [0.42 ; 12.57] |
| **CHG-T4** | 4/586 (0.682%) | 6.82 [1.86 ; 17.38] |
| **PVl-T1** | 15/576 (2.604%) | 26.04 [14.64 ; 42.58] |
| **PVl-T4** | 13/564 (2.305%) | 23.05 [12.32 ; 39.09] |

CI: Confidence interval.
