# Supplementary material for: Cost-effectiveness analysis of chlorhexidine-alcohol versus povidone iodine-alcohol solution in the prevention of intravascular-catheter-related bloodstream infections in France
Source: PLoS One. 2018 May 25;13(5):e0197747. doi: 10.1371/journal.pone.0197747 (PMC5969756; doi:10.1371/journal.pone.0197747)
Supplement: S5 File — (DOCX) [file pone.0197747.s008.docx]

## S5 File: Cost-Effectiveness base case analysis (ICU time horizon: 100 days – Global patient)

### Detailed results from the NH-SMC simulation for each patient group (ICU time horizon: 100 days – Global patient)

Note: After examination of transition probabilities in CLEAN database during 100 days (time horizon for base case analysis), 8 health states in CHG and PVI strategies were observed and retained for mathematical modeling.

Legend:

State1.noAE/noCRBSI/noCTnew;

State2.noAE/noCRBSI/CTnew;

State3.noAE/CRBSI/noCTnew;

State4.noAE/CRBSI/CTnew;

State5.AE/noCRBSI/noCTnew;

State6.G+S;

State7.Discharge;

State8.Death

R output:

Base case scenario results for CHG T1 group – Time Horizon: 100-days ICU

**## $prevalences$Expected (number of patients in each state)**

**## State 1 State 2 State 3 State 4 State 5**100 2.345515e-01 2.986414e-01 1.035132e-07 6.441799e-02 8.109308e-08

**## State 6 State 7 State 8 Total**100 4.368259e-02 389.5427 163.8160 554

**## $prevalences$`Expected percentages`(percentage of patients in each state)**

**## State 1 State 2 State 3 State 4 State 5**100 4.233781e-02 5.390638e-02 1.868470e-08 1.162780e-02 1.463774e-08

**## State 6 State 7 State 8**100 7.884943e-03 70.31456 29.56968


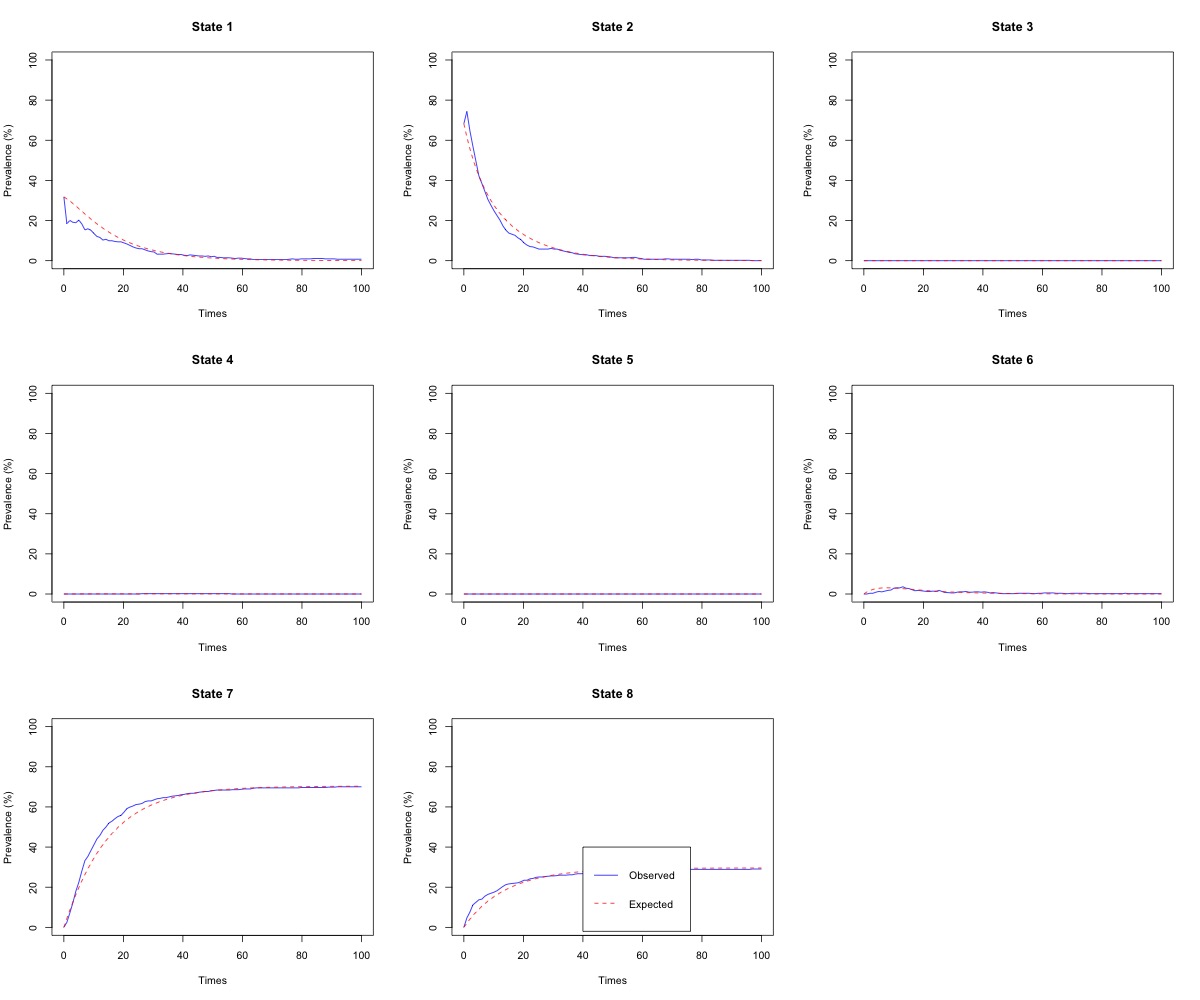


**## [1] "Estimated transition probability matrix, 100 days"**##
 **State 1 State 2 State 3 State 4 State 5**

State 1 0.0004141480 0.0005218447 1.825108e-10 8.560131e-05 1.423114e-10

State 2 0.0004276757 0.0005470812 1.888660e-10 1.305613e-04 1.482706e-10

State 3 0.0000000000 0.0000000000 0.000000e+00 0.000000e+00 0.000000e+00

State 4 0.0105356539 0.0198464017 4.956528e-09 3.601016e-02 4.671382e-09

State 5 0.0003070492 0.0003864532 1.352966e-10 6.119253e-05 1.054397e-10

State 6 0.0003070490 0.0003864529 1.352965e-10 6.119251e-05 1.054396e-10

State 7 0.0000000000 0.0000000000 0.000000e+00 0.000000e+00 0.000000e+00

State 8 0.0000000000 0.0000000000 0.000000e+00 0.000000e+00 0.000000e+00

**State 6 State 7 State 8**

State 1 7.701949e-05 0.7574664 0.2414350

State 2 7.970147e-05 0.6778534 0.3209616

State 3 0.000000e+00 1.0000000 0.0000000

State 4 2.091727e-03 0.6312164 0.3002996

State 5 5.709512e-05 0.7562528 0.2429354

State 6 5.709508e-05 0.7562529 0.2429354

State 7 0.000000e+00 1.0000000 0.0000000

State 8 0.000000e+00 0.0000000 1.0000000

**[1] "Expected Total Length of Stay (days) in each state for an individual between times [0,100]"**

**State 1 State 2 State 3 State 4 State 5**

8.886432e+00 4.950352e+00 1.503395e-06 2.999837e-02 1.639541e-06

**State 6 State 7 State 8**

6.344752e-01 6.522661e+01 2.027213e+01

**[1] "Expected number of visits, in each state, for an individual between times [0,100]"**

**State 1 State 2 State 3 State 4 State 5 State 6**

res 0.3079764 0.6746671 **0.0015033955 0.0010855802** 0.1504166 0.1504166

2.5% 0.2601520 0.5936940 0.0001947194 0.0001625962 0.1154792 0.1154792

97.5% 0.3560088 0.7546903 0.0086177730 0.0081743579 0.1967703 0.1967703

**State 7 State 8**

res 0.7574664 0.2414350

2.5% 0.7187051 0.2085157

97.5% 0.7905172 0.2798033

**## [1] "Expected mean cost (€) per individual during 100 days, per day and total, followed by confidence intervals: left and right bounds."**

**## Mean ci2.5% ci97.5%**## 0 1599.768845 1.599769e+03 1599.76884
## 1 1485.078375 1.461148e+03 1519.78209
## 2 1379.636075 1.337575e+03 1442.20079
## 3 1282.534410 1.226606e+03 1366.82639
## 4 1192.978887 1.126947e+03 1295.01800
## 5 1110.271040 1.035967e+03 1227.14116
## 6 1033.794229 9.535071e+02 1162.57165
## 7 963.001805 8.790486e+02 1101.08321
## 8 897.407217 8.110919e+02 1043.15604
## 9 836.575725 7.487591e+02 987.65829
## …

## 98 3.733997 9.341324e-01 60.20880
## 99 3.558059 8.686498e-01 59.24791
## 100 3.391513 8.065485e-01 58.30606
**## coutsTotal100 23797.725034 2.058395e+04 34330.75433**

Base case scenario results for CHG T4 group - Horizon: 100-days ICU

**## $prevalences$Expected (number of patients in each state)**

**## State 1 State 2 State 3 State 4 State 5**## 100 1.002502e-01 1.749267e-01 1.908378e-03 3.241160e-03 6.132784e-08

**## State 6 State 7 State 8 Total**## 100 8.623711e-02 423.7177 146.9158 571

**## $prevalences$`Expected percentages`(percentage of patients in each state)**

**## State 1 State 2 State 3 State 4 State 5**## 100 1.755695e-02 3.063515e-02 3.342169e-04 5.676288e-04 1.074043e-08

**## State 6 State 7 State 8**## 100 1.510282e-02 74.20625 25.72956


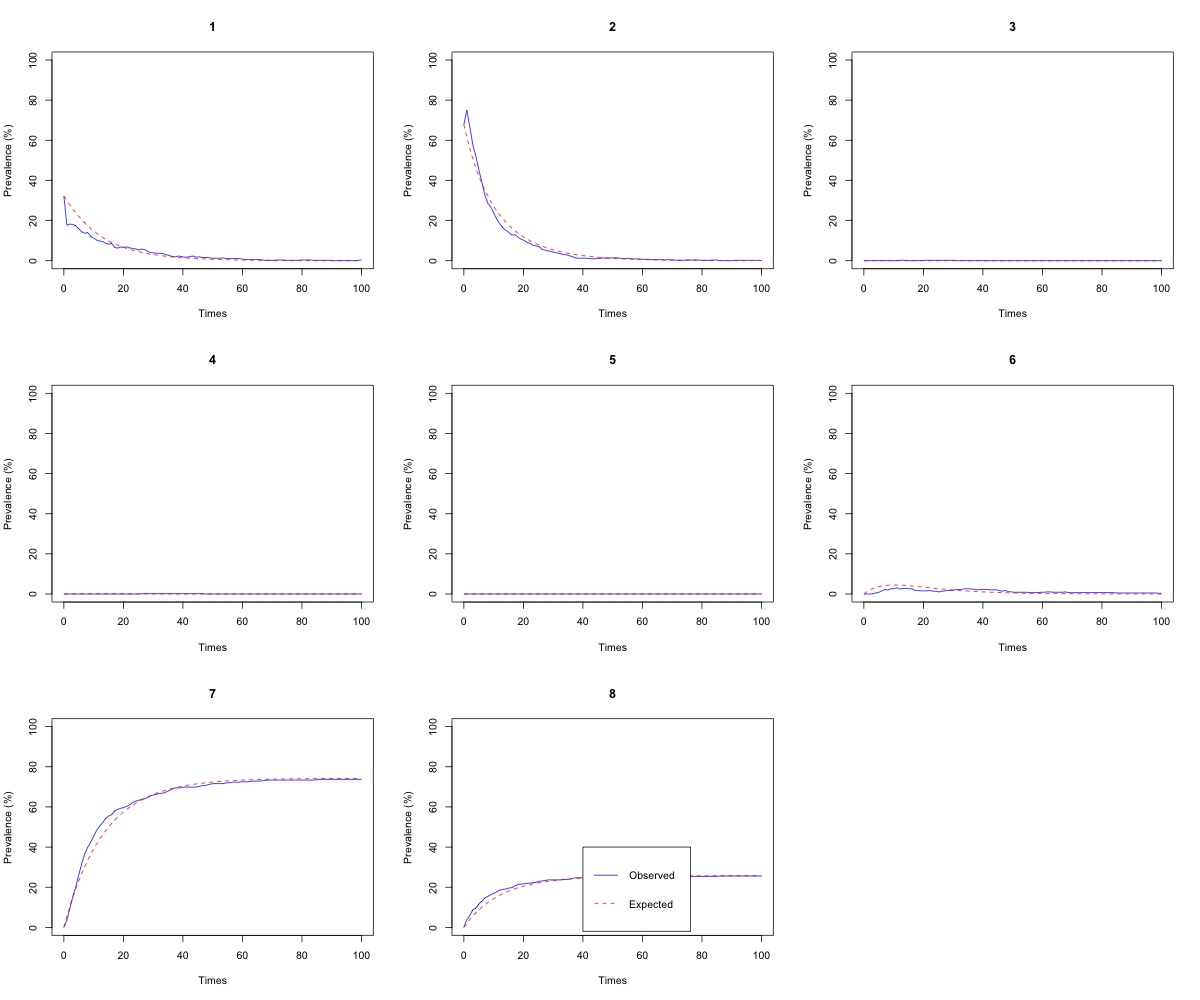


**## [1] "Estimated transition probability matrix, 100 days"**
##
**## 1 2 3 4 5**## 1 0.0001653084 0.0002885690 3.147886e-06 5.231186e-06 1.011653e-10
## 2 0.0001804481 0.0003148062 3.434541e-06 5.887912e-06 1.103706e-10
## 3 0.0001570425 0.0002741252 3.147569e-06 4.753212e-06 9.565396e-11
## 4 0.0006740117 0.0011044054 1.054841e-05 8.963318e-05 3.766473e-10
## 5 0.0002337813 0.0004086410 4.621049e-06 6.770884e-06 1.446650e-10
## 6 0.0002337812 0.0004086407 4.621046e-06 6.770879e-06 1.446648e-10
## 7 0.0000000000 0.0000000000 0.000000e+00 0.000000e+00 0.000000e+00
## 8 0.0000000000 0.0000000000 0.000000e+00 0.000000e+00 0.000000e+00
**## 6 7 8**## 1 0.0001422695 0.7844900 0.2149055
## 2 0.0001551925 0.7218902 0.2774500
## 3 0.0001336664 0.8920368 0.1073905
## 4 0.0004961280 0.7831289 0.2144964
## 5 0.0002062126 0.8460990 0.1530410
## 6 0.0002062124 0.8460990 0.1530410
## 7 0.0000000000 1.0000000 0.0000000
## 8 0.0000000000 0.0000000 1.0000000

**[1] "Expected Total Length of Stay in each state for an individual between times [0,100] "**

**## 1 2 3 4 5**## 6.691269e+00 4.877531e+00 1.727689e-02 2.304823e-02 1.719456e-06
**## 6 7 8**## 1.157351e+00 6.870618e+01 1.852734e+01

**[1] "Expected number of visits, in each state, for an individual between times [0,100]"**

**1 2 3 4 5 6**

res 0.3152679 0.7162449 0.0026615403 0.0021995587 0.1610036 0.1610036

2.5% 0.2652787 0.6294936 0.0007736653 0.0004902065 0.1243070 0.1243070

97.5% 0.3673796 0.7959918 0.0134729805 0.0088937124 0.2098352 0.2098352

**7 8**

res 0.7844900 0.2149055

2.5% 0.7458878 0.1811022

97.5% 0.8185362 0.2535064

**## [1] "Expected mean cost (€) per individual during 100 days, per day and total, followed by confidence intervals: left and right bounds."**

**## Mean ci2.5% ci97.5%**## 0 1577.1725 1577.1725 1577.1725
## 1 1458.6302 1429.3595 1506.1055
## 2 1349.7618 1298.7968 1433.2213
## 3 1249.6496 1182.9617 1358.5833
## 4 1157.4837 1078.6433 1287.9743
**## Mean ci2.5% ci97.5%**## 5 1072.5471 985.6293 1218.4738
## 6 994.2014 901.5544 1151.1481
## 7 921.8766 825.0042 1085.3904
## 8 855.0617 756.4388 1024.9402
## 9 793.2968 694.2201 967.9964
##...

## 98 1.229786 5.570676e-01 15.86606
## 99 1.143835 5.150919e-01 15.28281
## 100 1.063889 4.760434e-01 14.72902
**## coutsTotal100 21822.3199 1.863496e+04 29700.65665**

Base case scenario results for PVI T1 group - Horizon: 100-days ICU

**## $prevalences$Expected (number of patients in each state)**

**## State 1 State 2 State 3 State 4 State 5**## 100 0.16870242 0.29957754 0.0063365166 0.0027565892 1.082955e-07

**## State 6 State 7 State 8 Total**## 100 0.10115114 401.5819 161.8396 564

**## $prevalences$`Expected percentages`(percentage of patients in each state)**

**## State 1 State 2 State 3 State 4 State 5**## 100 0.029911777 0.053116585 1.123496e-03 4.887570e-04 1.920132e-08

**## State 6 State 7 State 8**## 100 0.017934600 71.20247 28.69496


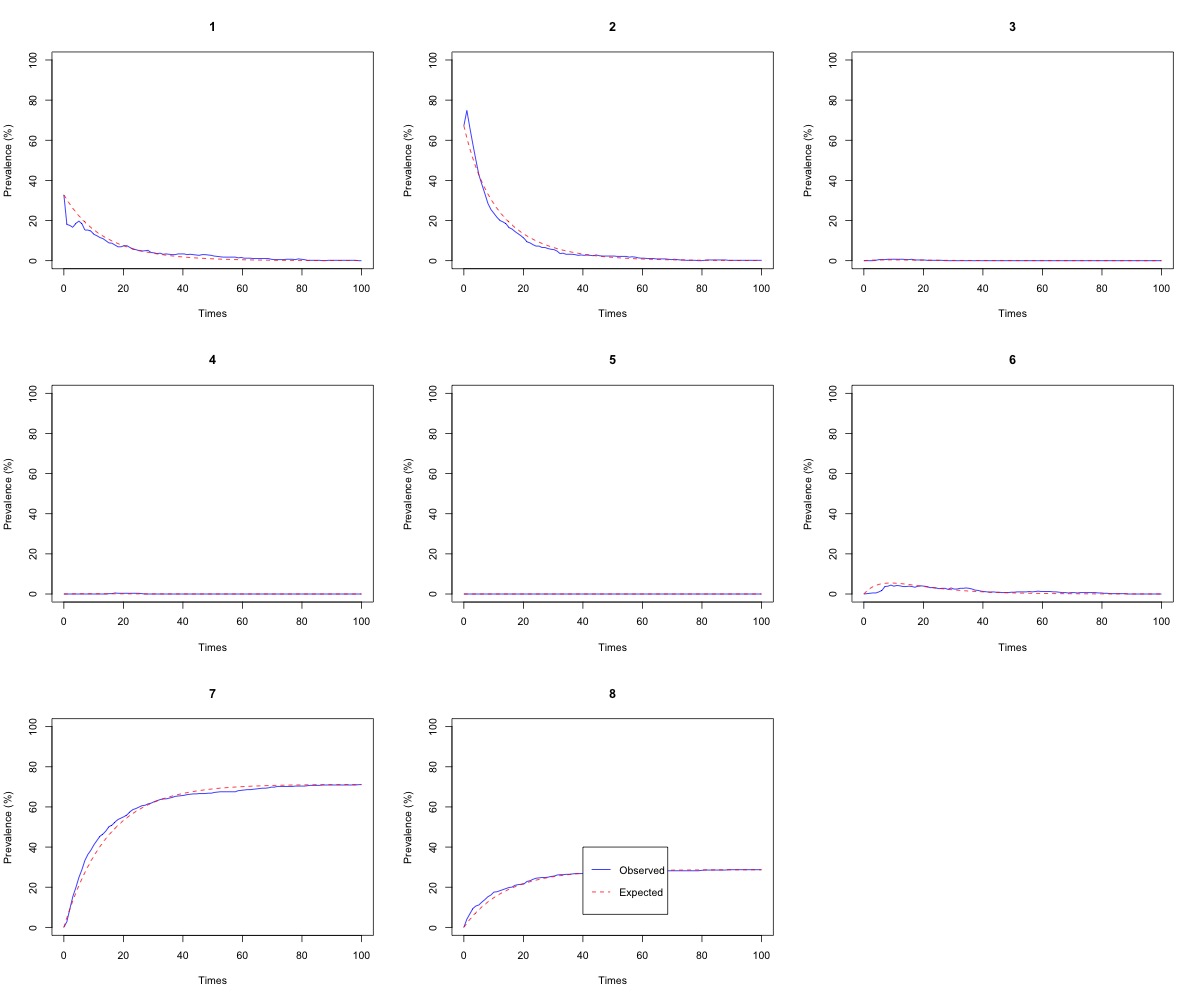


**## [1] "Estimated transition probability matrix, 100 days"
##
## 1 2 3 4 5**## 1 0.0002737906 0.0004861913 1.028366e-05 4.473456e-06 1.757536e-10
## 2 0.0003114806 0.0005531191 1.169931e-05 5.089709e-06 1.999500e-10
## 3 0.0003390481 0.0006020798 1.273968e-05 5.538093e-06 2.176334e-10
## 4 0.0004855690 0.0008622839 1.823651e-05 7.943931e-06 3.116849e-10
## 5 0.0003192904 0.0005669766 1.199267e-05 5.219932e-06 2.049809e-10
## 6 0.0003192902 0.0005669762 1.199266e-05 5.219928e-06 2.049807e-10
## 7 0.0000000000 0.0000000000 0.000000e+00 0.000000e+00 0.000000e+00
## 8 0.0000000000 0.0000000000 0.000000e+00 0.000000e+00 0.000000e+00
**## 6 7 8**## 1 0.0001641542 0.7680275 0.2310336
## 2 0.0001867615 0.6846882 0.3142437
## 3 0.0002032329 0.7931069 0.2057304
## 4 0.0002910506 0.7050683 0.2932666
## 5 0.0001915230 0.7513147 0.2475903
## 6 0.0001915228 0.7513147 0.2475903
## 7 0.0000000000 1.0000000 0.0000000
## 8 0.0000000000 0.0000000 1.0000000

**[1] "Expected Total Length of Stay in each state for an individual between times [0,100] "**

**## 1 2 3 4 5**## 6.979218e+00 5.281915e+00 9.260848e-02 2.632880e-02 2.146182e-06
**## 6 7 8**## 1.193869e+00 6.689506e+01 1.953100e+01

**[1] "Expected number of visits, in each state, for an individual between times [0,100]"**

**## 1 2 3 4 5 6**## res 0.3484878 0.7566631 0.01757669 0.004790418 0.2031719 0.2031719
## 2.5% 0.2989613 0.6734627 0.01004481 0.001889737 0.1639783 0.1639783
## 97.5% 0.4106787 0.8488544 0.03817883 0.016641934 0.2505170 0.2505170
**## 7 8**## res 0.7680275 0.2310336
## 2.5% 0.7269172 0.1978317
## 97.5% 0.8013203 0.2720146

**## [1] "Expected mean cost (€) per individual during 100 days, per day and total, followed by confidence intervals: left and right bounds."**

**## Mean ci2.5% ci97.5%**## 0 1590.6126 1590.6126 1590.6126
## 1 1496.1343 1460.7587 1547.9403
## 2 1405.9669 1344.1194 1494.7924
## 3 1320.1662 1238.4602 1438.6395
## 4 1238.7254 1142.1085 1377.9507
## 5 1161.5895 1054.2211 1315.3224
## 6 1088.6662 975.1702 1258.0536
## 7 1019.8358 901.2726 1199.6768
## 8 954.9588 832.4217 1142.6597
## 9 893.8819 770.2200 1082.8271
##...

## 98 2.013234 1.007736e+00 4.826409
## 99 1.879579 9.354438e-01 4.530711
## 100 1.754796 8.684734e-01 4.261036
**## coutsTotal100 24873.845 2.101111e+04 31678.318363**

Base case scenario results for PVI T4 group - Horizon: 100-days ICU

**## $prevalences$Expected (number of patients in each state)**

**## State 1 State 2 State 3 State 4 State 5**## 100 1.915665e-01 3.084615e-01 7.214600e-04 5.270692e-03 1.477991e-07

**## State 6 State 7 State 8 Total**
## 100 2.019878e-01 388.4830 156.8090 546

**## $prevalences$`Expected percentages`(percentage of patients in each state)**

**## State 1 State 2 State 3 State 4 State 5**## 100 0.0350854453 0.0564947773 1.321355e-04 9.653283e-04 2.706944e-08

**## State 6 State 7 State 8**## 100 0.0369940980 71.15073 28.71959


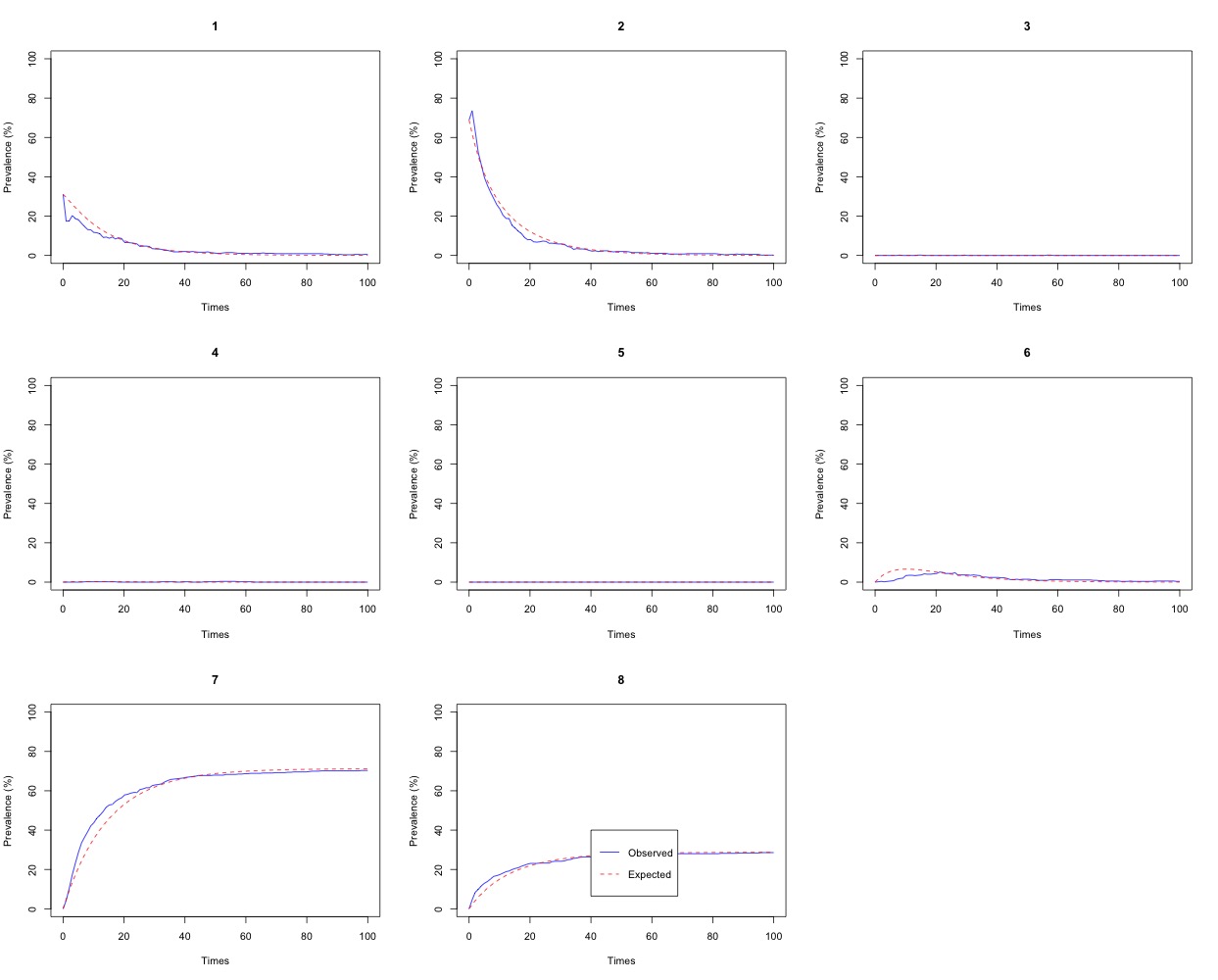


**## [1] "Estimated transition probability matrix, 100 days"
##
## 1 2 3 4 5**## 1 0.0003359929 0.0005410011 1.264816e-06 9.242679e-06 2.591480e-10
## 2 0.0003575738 0.0005757747 1.346918e-06 9.838928e-06 2.759148e-10
## 3 0.0003150427 0.0005072828 1.186459e-06 8.667909e-06 2.430610e-10
## 4 0.0004232148 0.0006814848 1.594507e-06 1.164927e-05 3.266138e-10
## 5 0.0004821802 0.0007766000 1.822566e-06 1.328555e-05 3.729496e-10
## 6 0.0004821799 0.0007765995 1.822564e-06 1.328554e-05 3.729493e-10
## 7 0.0000000000 0.0000000000 0.000000e+00 0.000000e+00 0.000000e+00
## 8 0.0000000000 0.0000000000 0.000000e+00 0.000000e+00 0.000000e+00
**## 6 7 8**## 1 0.0003539893 0.7695806 0.2291779
## 2 0.0003771532 0.6852508 0.3134275
## 3 0.0003321687 0.7517082 0.2471274
## 4 0.0004465521 0.6583967 0.3400388
## 5 0.0005116892 0.7596581 0.2385563
## 6 0.0005116889 0.7596581 0.2385563
## 7 0.0000000000 1.0000000 0.0000000
## 8 0.0000000000 0.0000000 1.0000000

**[1] "Expected Total Length of Stay in each state for an individual between times [0,100] "**

**## 1 2 3 4 5**## 7.364896e+00 5.154162e+00 1.311806e-02 5.019776e-02 2.336123e-06
**## 6 7 8**## 1.614992e+00 6.656536e+01 1.923727e+01

**[1] "Expected number of visits, in each state, for an individual between times [0,100]"**

**## 1 2 3 4 5 6**## res 0.3364113 0.7900335 0.013108179 0.009799080 0.2143911 0.2143911
## 2.5% 0.2877113 0.7081256 0.006352126 0.005331494 0.1736784 0.1736784
## 97.5% 0.3940857 0.8814961 0.035631743 0.021575512 0.2714106 0.2714106
**## 7 8**## res 0.7695806 0.2291779
## 2.5% 0.7289344 0.1959974
## 97.5% 0.8030879 0.2695348

**## [1] "Expected mean cost (€) per individual during 100 days, per day and total, followed by confidence intervals: left and right bounds."**

**## Mean ci2.5% ci97.5%**## 0 1619.3122 1619.3122 1619.3122
## 1 1511.3404 1475.5283 1562.8530
## 2 1408.5333 1346.5665 1492.4201
## 3 1312.1282 1231.4510 1418.1269
## 4 1222.2495 1128.1470 1343.9457
**## Mean ci2.5% ci97.5%**## 5 1138.6367 1034.3758 1271.3832
## 6 1060.9106 949.5140 1199.4546
## 7 988.6691 872.6282 1132.0440
## 8 921.5213 803.9350 1068.0626
## 9 859.0986 740.7754 1006.1318
##...

## 98 2.325498 1.110744e+00 4.537680
## 99 2.178079 1.033495e+00 4.270863
## 100 2.040007 9.616028e-01 4.019694
**## coutsTotal100 24201.191247 2.050724e+04 29135.741633**
