# Supplementary material for: Cost-effectiveness analysis of chlorhexidine-alcohol versus povidone iodine-alcohol solution in the prevention of intravascular-catheter-related bloodstream infections in France
Source: PLoS One. 2018 May 25;13(5):e0197747. doi: 10.1371/journal.pone.0197747 (PMC5969756; doi:10.1371/journal.pone.0197747)
Supplement: S6 File — (DOCX) [file pone.0197747.s009.docx]

## S6 File: Hazard ratios (HR) and 95% confidence intervals (95%CI) for each subgroup

Hazard ratios (HR) and 95% confidence intervals (95%CI) represent the effect for each subgroup. The p-value tests the heterogeneity of CHG-gel dressing effect between subgroups. A p-value >0.05 indicates the absence of significant differences of effects between subgroups.
